# Reinforcement Learning Algorithms for Autonomous Mission Accomplishment by Unmanned Aerial Vehicles: A Comparative View with DQN, SARSA and A2C

**DOI:** 10.3390/s23219013

**Published:** 2023-11-06

**Authors:** Gonzalo Aguilar Jiménez, Arturo de la Escalera Hueso, Maria J. Gómez-Silva

**Affiliations:** 1 Dana SAC Spain, S.A., Dana Off-Highway, C/Abedul S/N, Pol. Ind. Los Huertecillos, 28350 Ciempozuelos, Madrid, Spain; 2 Intelligent Systems Lab, Universidad Carlos III de Madrid, Avda de la Universidad 30, 28911 Leganés, Madrid, Spain; escalera@ing.uc3m.es; 3 Department of Computer Architecture and Automation, Facultad de Ciencias Físicas, Universidad Complutense de Madrid, Plaza Ciencias 1, 28040 Madrid, Spain; mgomez77@ucm.es

**Keywords:** reinforcement learning, DQN, SARSA, A2C, drone, quadrotor, UAV

## Abstract

Unmanned aerial vehicles (UAV) can be controlled in diverse ways. One of the most common is through artificial intelligence (AI), which comprises different methods, such as reinforcement learning (RL). The article aims to provide a comparison of three RL algorithms—DQN as the benchmark, SARSA as a same-family algorithm, and A2C as a different-structure one—to address the problem of a UAV navigating from departure point A to endpoint B while avoiding obstacles and, simultaneously, using the least possible time and flying the shortest distance. Under fixed premises, this investigation provides the results of the performances obtained for this activity. A neighborhood environment was selected because it is likely one of the most common areas of use for commercial drones. Taking DQN as the benchmark and not having previous knowledge of the behavior of SARSA or A2C in the employed environment, the comparison outcomes showed that DQN was the only one achieving the target. At the same time, SARSA and A2C did not. However, a deeper analysis of the results led to the conclusion that a fine-tuning of A2C could overcome the performance of DQN under certain conditions, demonstrating a greater speed at maximum finding with a more straightforward structure.

## 1. Introduction

Because of the modern tendency to automate, the advances in drone navigation, and the robust capabilities and reduced size of the onboard processors, it has been possible during recent years to design small UAVs. They feature functions like photo and video recording, object recognition, tracking, and autonomous flight, among various other algorithms that provide drones with artificial intelligence capabilities.

Machine learning (ML) [1], which is a type of artificial intelligence (AI), emerged from the necessity to solve complex decision-making problems when direct programming became too tedious. It is a procedure intended to make predictions using different algorithms based on data probability and statistics [2] without a specific designed. It comprises approaches like supervised learning (SL) [1,3], unsupervised learning (UL) [4,5,6], and reinforcement learning (RL) [7]. The first is used when the provided data are perfectly labeled and the software must foresee the outcome based on a previously guided learning process. The second, UL, is used when data are not categorized and the algorithm must cluster these data to present a result. Finally, RL is employed when no preliminary data are provided and the algorithm must learn without previous knowledge, which is achieved by generating and discarding the data acquired based on a policy aiming to look for the best final return, i.e., the reward.

RL resulted from the necessity of solving Markov decision processes that, due to their complexity, required an exact and well-modeled classical dynamic programming (DP) [8] approach. Initially, RL started with temporal difference (TD) learning [9], state-action-reward-state-action (SARSA) [10], and Q-Learning [11] based on value estimation. However, as the difficulty increased, users solved such problems using non-dedicated solutions, either improving existing ones like deep Q-Network (DQN) [12] vs. Q-Learning or creating new ones, like the reinforce algorithm [13], based on the policy gradient. The combination of both models, i.e., value estimation and policy gradient ones, gave birth to actor–critic methods, like advantage actor-critic (A2C) [14]. 

For the process of choosing the best algorithm suited to a particular project, this work compared three RL algorithms to solve a problem involving a drone traveling autonomously from start point A (0,0) to endpoint B (35,50). Several parameters are involved in a drone’s flight; therefore, an algorithm might be applicable depending on the problem, i.e., navigation, control, attitude, takeoff, landing, hovering, obstacle avoidance, or trajectory planning. In particular, there is a suitable algorithm for each activity, which can be noticed if attention is paid to the importance of UAV channels in path planning [15,16]. Since there is not much literature on a similar approach to this problem, giving a benchmark to compare with is crucial, helping to measure the results of the other algorithms with its own. 

This work uses DQN as a reference, primarily for being one of the first RL algorithms to combine RL with deep neural networks (DNN), resulting in deep RL (DRL). Secondly, several authors have employed it either for a similar purpose with a more accessible environment [17] or in the same domain as the one used, but for a different purpose [18]. In addition, it has been used for a different environment and purpose while maintaining the overall idea of using one algorithm for a work involving multitasking [18].

Some authors have applied DQN to UAVs, using the method in similar applications. Kjell [17] used DQN in a straightforward scenario, where the only objects were a series of columns of exact dimensions, distributed homogeneously. Some might wonder whether DQN, with its identical structure, could fit this application in any other environment or if any change would be needed. Microsoft [18] has provided two approaches: the first is using a DQN agent to identify and follow the powerlines in a mountainous environment. The UAV must fly along the lines, learning to distinguish them from the other objects. Given that it develops a single-task work, this DQN structure fits their requirements. The second is employing DQN for a self-driven car in the neighborhood environment, the same environment as that of this work. The vehicle’s purpose would be to autonomously move on the roads, avoiding other vehicles and turning when needed while regulating the speed and traveling from a departure point to an endpoint without crashing. 

Given that DQN is a deep RL method, the algorithm combines the features of a deep neural network (DNN) and RL, which is equivalent to saying that it needs a neuron structure capable of learning and a state-action-reward threesome that satisfies the learning activity of the neurons. The number of neurons required and actions to be performed can be called into question depending on the previous ones. Also, it might be questioned how challenging the environment or the activity can become before thinking about an alternative; whether the learning process is too long to look for a different algorithm; and which features would be required in an algorithm to accomplish its goal better than DQN.

This work compares DQN with two other algorithms to evaluate the performances of each of them when undertaking the same activity. It also questions the suitability of DQN for UAVs’ autonomous missions. These two different algorithms have been selected according to their features based on previous works. There are algorithms intended for single tasks, like DQN and SARSA, and others for multitasking activities, like A2C.

The second algorithm to be compared is SARSA, which has already been introduced. It is a temporal difference (TD) algorithm, such as DQN; therefore, it is interesting to compare it with DQN for the required task of checking variations within the same family. Additionally, SARSA applies to multiple tasking projects [14], as it has been demonstrated that genetically regulated neuromodulation (GRNM) SARSA, a variable-parameters SARSA, can exceed the expectations of standard SARSA with fixed parameters. Therefore, if GRNM SARSA excels at multitasking problems by reductio ad absurdum, it means that SARSA can also solve multitasking duties. However, it is essential to mention that SARSA serves the purpose of family comparison because other multiple choices are more appropriate when considering multitasking issues. 

Multitasking duties need specific features to be addressed, such as those seen in Table 1, extracted from [19]. Some of the algorithms capable of dealing with the duties/issues of multitasking are also shown in the table, which indicates the features that can be partially or entirely solved with each algorithm.

The last of the algorithms to be compared is the asynchronous advantage actor–critic (A3C), proposed by DeepMind based on parallel training, where various agents (workers) perform in parallel in the same environment and have different experiences [20]. During the training, multiple workers update the global information asynchronously, and due to the specific knowledge of the same environment, the approach provides a practical exploration and the data sample efficiency [21]. A3C is founded on the actor–critic method, in which the actor performs actions and the critic evaluates the results of the actions taken by the actor [20]. The critic approximates the value function, an action value according to the Q-value or a state value. The advantage, A, states that selecting an action over the other possibilities is better. This advantage actor–critic approach can be synchronous or asynchronous; that is, advantage actor–critic (A2C) or A3C. A2C is the synchronous version of A3C, where the implementation waits for every actor to finish its work before updating. One of the advantages obtained with A2C is the higher effectivity on GPUs (graphic processing units), which allows for higher efficiency with large batches [22].

The contribution made by comparing the three algorithms, DQN, SARSA, and A2C, allows us see the suitability of the benchmark, DQN, and identify whether the family of multitasking algorithms performs better at UAV autonomous mission accomplishment than the single-tasking algorithms. The comparison with the same “family” (SARSA) strengthens DQN as the benchmark within its group. The comparison with A2C continues widening the position as a benchmark within other families in RL algorithms. The data acquisition was completed using a stereo camera, and the data were analyzed and engineered below to output the results.

This article presents a critical review that reveals that the performance achieved by each method depends on the evaluation criterion and the specifications of the task being addressed. Given the wide variety and disparity of evaluation criteria and metrics in the literature, this paper contributes a systematic comparison of methods to provide guidelines for future research and implementations.

The analysis of the results led to the conclusion that under the premises given, the benchmark DQN showed the best performance. This remained undisputed; however, A2C showed behavior that will induce further study, since, from the results, it seems that the data sample efficiency feature had a good result in the neighborhood environment due to an early maximum finding with fewer parameters. Establishing a unique environment to unify criteria and validate the performance comparison is crucial.

The rest of the manuscript is outlined as follows. Section 2 presents the reasons to address the problem of an autonomous UAV undertaking a mission involving traveling from point A to point B; the methods used to approach the research; and the reliability and validity criteria. Section 3 contains a short description of the meaning of the analyzed parameters to compare the performance of the algorithms and the obtained results. Section 4 discusses the results, providing information about the comparison and the results’ implications. Finally, Section 5 ends the paper by providing evidence and details of the conclusions expressed previously about the utility of each algorithm. 

## 2. Materials and Methods

Section 2 outlines the development of the project. It provides information about the tools, programs, and premises chosen for the simulation and data collection. It also explains the type of environment, i.e., an urban neighborhood, and how the simulator, AirSim, works. It also provides information regarding the selected algorithms, with their equations and pseudocodes. Finally, it explains the outputs used for the data analysis. 

This work collects recent investigations into different methods by which to make a drone fly autonomously, avoiding obstacles, which is possible thanks to sensors like RGB cameras. These, for example, can estimate the attributes of a reef or a maritime forest; in ref. [23], the attributes of the canopy of a beech forest were estimated using an RGB camera with a fixed-wing UAV. These goals can also be accomplished thanks to infrared cameras or LIDAR (LIght Detection And Ranging), as well as algorithms like DQN or DDPG (deep deterministic policy gradient). To cite some references regarding DDPG, we found an experiment comparing DDPG with DQN on a UAV, where the training experiments showed that DDPG has better adaptability features in dynamic and complex environments [24]. This method was utilized to reduce the dependence of flight autonomy on the controller model’s design, which was proportional–integral–derivative (PID), using a simulation to demonstrate the convergence speed learning ratio when the dynamic model was unknown [25].

Regarding UAV control with DDPG, the algorithm was extended to “Multiple Experience Pools DDPG (MEP-DDPG)”, designed under a “predictive model control and simulated annealing to generate expert experience” and showing an improvement of over 20% in unknown surroundings [26].

During path planning for UAVs, when tracking a ground target, an improved algorithm based on DDPG was used, showing efficient simulated results in terms of approximating the target while avoiding obstacles [27]. A similar study proposed that DDPG is made for “UAV air combat autonomous maneuver decision” instead of DQN—the current state of the art—and would improve learning efficiency [28].

### 2.1. Experimental Environment

Many factors take part in the process of flying from a start point A to a target point B. Some depend on the drone configuration, either software, like control algorithms, or hardware, i.e., size, weight, or sensors; others rely on the environment. Like the atmosphere-reliant factors, others are independent because UAVs cannot influence them, but adapt the flight mode algorithm to these conditions. For example, the flight path may vary regarding the wind loads and direction, and the distance to objects may differ depending on the camera’s characteristics and adaptability to different visibility conditions.

**Figure 1:**
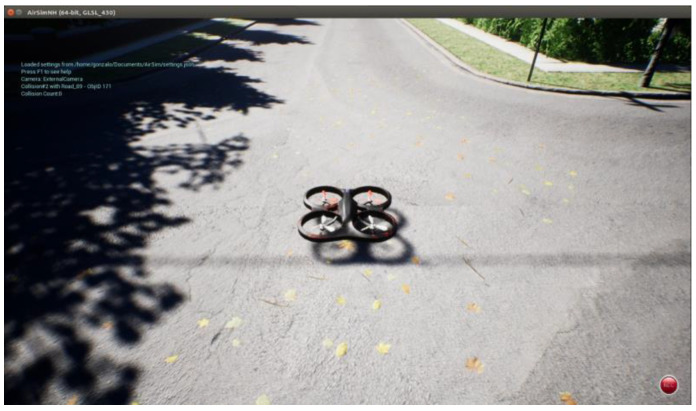
Departure point A (0,0). Obtained from AirSim. Crossroad of the neighborhood in Unreal Engine 4.

This research concerns a simulated UAV taking off from the center of a virtual neighborhood’s crossroad in a standard western city. See Figure 1. 

**Figure 2:**
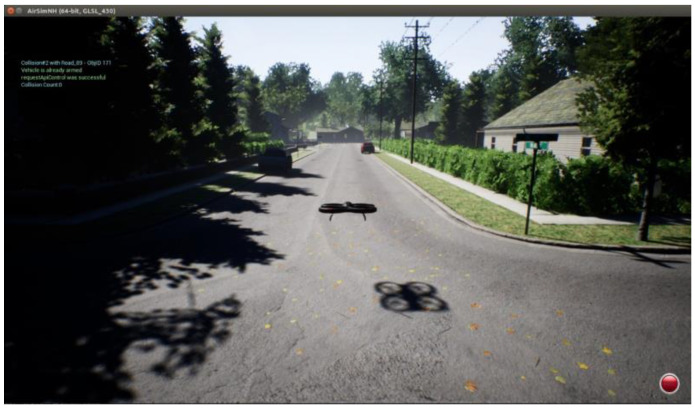
View of components of the environment. Obtained from AirSim. Objects found are trees, houses, traffic signs, and many bushes. These objects affect the trajectory planning.

There are realistic objects, such as cars, houses, trees, and bushes, in this environment, which are the obstacles to avoid. See Figure 2.

**Figure 3:**
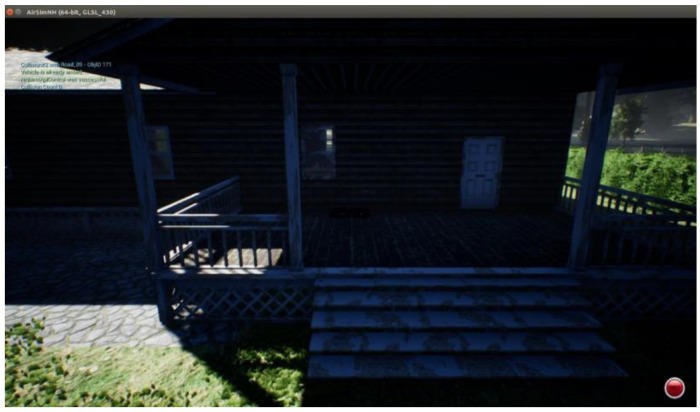
Different light intensities. Proof of the many distinct ways an object can look depending on the darkness. Light intensity affects the data observation and processing.

**Figure 4:**
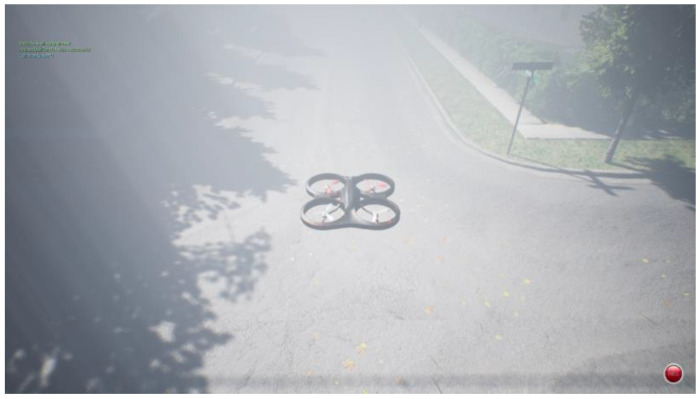
Foggy conditions, obtained from AirSim. Weather conditions like wind might also affect trajectory planning.

The atmospheric conditions are variable; thus, changing the light and visibility, i.e., sunlight, shadows, or fog, affects sensors and loads, i.e., wind speed, on the drone, multiplying the study options. See Figure 3 and Figure 4.

### 2.2. Method Explanation

A comparison of the results obtained by DQN, SARSA, and A2C is presented. Although some of the reasons for choosing those three algorithms have been introduced, a more detailed discussion is presented in this section.

DQN is the baseline algorithm implemented for UAV [17,18]; therefore, it is essential to this work, since it is the one to “beat”. The other methods may be better or worse for different applications, but this work references their results to the DQN outcomes. 

SARSA was included because it is closely connected to Q-Learning; thus, it is necessary to introduce Q-learning and its relationship with DQN. Q-Learning is a robust algorithm, but is also weak due to its lack of generality, so it cannot predict the proper action for future states. DQN solved the Q-Learning problem by adding NNs that allowed the method to foresee the future best action.

Regarding how SARSA solves the Q-learning deficit, it utilizes the behavior policy, usually *ε*-greedy, to choose the following action, then updates the Q-value with the next state, *s*, and subsequent action *a*, as the expected future return. However, Q-learning updates the future return using the *max Q* operator, which is applied to the next state and current action. When using an *ε*-greedy policy for SARSA, as in this project, the learning process becomes a random exploration of the environment before approaching the algorithm’s convergence, and this would occur over an infinite time.

Knowing the similitudes between SARSA and Q-Learning (for extension, also to DQN), both equations can help to visualize the explanation. Firstly, the SARSA Equation (1), and then the Q-Learning Equation (2), are presented.

(1) $$Q\left(s_{t},\;a_{t}\right)\overset{}{\leftarrow }Q\left(s_{t},\;a_{t}\right)+\alpha \left[\;r_{t+1}+\gamma Q\left(s_{t+1},\;a_{t+1}\right)-Q\left(s_{t},\;a_{t}\right)\right]$$

(2) $$Q\left(s_{t},\;a_{t}\right)\;\overset{}{\leftarrow }Q\left(s_{t},\;a_{t}\right)+\alpha \left[\;r_{t+1}+\gamma \underset{a^{\prime }}{\max}Q\left(s_{t+1},\;a^{\prime }\right)-Q\left(s_{t},\;a_{t}\right)\right]$$

where *s_t_, a_t_,* and *r_t_* are the state, action, and reward at the time *t,* and *γ* is the discount factor. Both equations are similar. Q-Learning obtained the estimate after assessing all possible future actions, no matter the previous one. SARSA took the same action used to update the forecast. Some of the pros and cons of Q-Learning and SARSA are listed below:

- Q-Learning acquired the best policy, whereas SARSA attained a semi-optimal strategy at the time of discovery, which obliged us to determine an excellent rule with which to decide a policy to degenerate ϵ in ϵ-greedy, which can turn parameter tuning into a challenging task.
- SARSA had lower per-sample variance than Q-Learning (and thus DQN), suffering fewer converging problems, making it more difficult for the latter to train.
- SARSA is more conventional than Q-Learning (for extension, DQN) because it allows for possible penalties from different exploratory moves. For example, if the hazard of a tremendous adverse recompense near the best route existed, SARSA would avoid the dangerous ideal track and only gradually absorb how to utilize it once a few exploration hyperparameters were left. However, Q-Learning would try to activate that reward during exploration.

Advantage actor-critic (A2C) is part of a different group of algorithms. On the one hand, there is an actor, which is one neural network representing a policy that receives a state and gives actions. Conversely, the critic works as a value function, as DQN does. This critic evaluates how well the actor performs and improves the policy, if possible. This improvement uses a policy gradient to maximize the critic’s value estimates by refining the actor policy. The latter is a reason to think that A2C could be a DRL algorithm capable of overtaking the performance of DQN. 

The A2C network equation below (3) shows the inclusion of the Q-Learning algorithm in the advantage. The Q-Learning aspect denotes an NN through the *w* parameter indication:

(3) $$\nabla_{\theta }J\left(\theta \right)\;\sim \sum_{t=0}^{T-1}\nabla_{\theta }\log\pi_{\theta }(a_{t}|s_{t})(r_{t+1}+\gamma V_{v}\left(s_{t+1}\right)-V_{v}\left(s_{t}\right))=\;\sum_{t=0}^{T-1}\nabla_{\theta }\log\pi_{\theta }(a_{t}|s_{t})A\left(s_{t},\;a_{t}\right)$$

From the Bellman optimality equation [29], we can rewrite the advantage as (4):

(4) $$A\left(s_{t},\;a_{t}\right)=Q_{w}\left(s_{t},\;a_{t}\right)-V_{v}\left(s_{t}\right)$$

For a better understanding of the terms comprising the A2C equation, a short description is provided below:

o $\nabla_{\theta }J\left(\theta \right)$: Policy gradient
o $A\left(s_{t},\;a_{t}\right)$: Advantage value
o $Q_{w}\left(s_{t},\;a_{t}\right)$: Q value learned by parameterizing (*w*) with a NN
o $V_{v}\left(s_{t}\right)$: V value learned by parameterizing (*v*) with the NN

The comparison was made using OpenAI Gym, “a toolkit for developing and comparing RL algorithms. It supports teaching agents everything from walking to playing games” [30]. 

To apply a DRL formula to the task presented, it is necessary to analyze images captured by a sensor. In this project, a stereo camera caught images using depth measuring. These images were internally processed, analyzed, and compared by the algorithm, and then the neurons made decisions that led to a better or worse overall performance. 

Firstly, knowing the system’s features in order to distinguish which DRL method could fit the application was essential. The system characteristics analysis led to the following results:

- Observation space: Box shape (30, 100) represents an n-dimensional space called an array (30, 100). The algorithm works with tensors (part of images) of the above shape, deducing it as a discrete observation space. Previous works utilized this shape [17], so our decision was to do the same.
- Action space: The action space is the number of possible decisions the actor can make every time it receives a new observation space. It is defined as discrete if there are only a few measurable possible actions or continuous if there are infinite options. The action space is discrete, with three (3) actions for the selected actor: turning right, turning left, and moving forward.

It would be expected to add a variable height, *z*; to go backward, *-x*; or even to move right and left, *+y* and *-y*.

For apprenticeship reasons, *z* would be constant because, using a variable z, the algorithm would quickly learn that the easiest way to avoid obstacles is to travel up over them and then move to the target point B. If that happened, the study would be invalid.

Regarding the possibility of going backward, there are two reasons:

- Simplicity: because *-x* is the same as turning 180° and then moving *+x* (5), according to the AirSim code structure:

(5) $$\left|x\right|=\left|v\right|\times t$$

o *x*: linear displacement
o *v*: linear velocity
o *t*: time

- Due to the camera sensor’s location, which is at the front, another camera would be necessary at the rear side of the UAV to move backward.

Furthermore, moving right and left would complicate the processing due to many unnecessary variables. It would be the same as turning +90° or −90° and moving forward.

Finally, the process of turning right or left, according to the Formula (6), is given below:

(6) ϑ=ω×t

o *ϑ*: angular displacement
o *ω*: angular velocity
o *t*: time

Considering |*ω|* as a constant, positive *+ω,* or negative −*ω,* to indicate right or left, respectively, the reader can see that *t* is the only variable. Hence, the angle variation depends only on for how long the algorithm commands the maneuver.

Thus, to summarize the system has three variables—*+x*, *t*, and the sign of *ω*—defining the three possible movements. See Equations (7)–(9).

(7) t×ω=turn right

(8) t×−ω=turn left

(9) +x=forward

In the description, the system is illustrated as discrete in both observation and action spaces. Still, it is essential to explain that, for this application, every space is discrete if it is not continuous. Therefore, it needs to be real-valued, which is not the case.

With the previous paragraph’s data, it is noticeable that the selected algorithms can work with the discrete type for their observation and action spaces.

As a short compilation of the chosen RL methods, here are some quick details:

- Since the algorithms use NNs, the inputs are the observation spaces, and the outputs are the actions taken.
- Only A2C comprises an NN for the advantage, the actor and critic, an agent, and a supervisor.

### 2.3. Comparative Study Methodology

This section addresses the methodological approaches, the type of knowledge produced, and the data collected in order to implement the method.

After observing the state of the art, the necessity of an objective comparison of the benchmark DQN with other methods was detected. Extensive previous work in the field exists, but none has contrasted different algorithms for a specific task or with fixed hypotheses. 

Therefore, the goal was to contribute to solving the newly identified issue of recognizing the current best RL algorithm within these three algorithms to control a drone, to avoid obstacles using a stereo camera; and, if necessary, to update the benchmark.

Finally, regarding the hypotheses used, there is consideration regarding the previous work on RL algorithms, which performed the best in drones that excelled with A2C or SARSA networks. 

The three descriptive paragraphs above help to explain this type of research:

- It is an applied method, because it solves a practical problem with a new set of techniques;
- It is exploratory, since it is a recently identified issue and intends to contribute new answers;
- Combining the previous hypothesis regarding RL algorithms and developing an innovative one is a partially inductive and partially deductive approach.

### 2.4. Data Collection

This section describes the research details, the tools and procedures used to gather data, and the criteria used to select the participating DRL algorithms.

The introduction briefly explains different tools, like AirSim and UE4. A preliminary notion of what will be described below, i.e., how to reproduce the system, is established. Thus, interested researchers may replicate and improve upon this work.

This project utilized UE4 for environmental reproduction because AirSim, the simulator selected for the research, was initially designed for it. However, later, Microsoft implemented it for Unity.

There are some alternatives to AirSim for drone simulation, like Gazebo. However, since AirSim can work on a HITL simulation, the 3D graphics should be pretty realistic and extendable to future works. Besides, the Microsoft team has also developed the API [29] to connect AirSim with PX4 (Pixhawk SITL) and directly with Pixhawk (HITL), allowing the control of a physical drone either in SITL or HITL with the QGroundControl (QGC, version 4.0) [31], which is software that, in the case of failure of the NN, can take control of the drone and allow for human operation.

OpenAI Gym, which has already been introduced, was used to execute this work. It provided the environment in which to train the algorithm without creating it, allowing users to focus only on the NN’s design. OpenAI Gym enables the use of several different environments produced by Microsoft. These diverse environments permit the training and testing of algorithms in distinct situations. In Ref. [32], AirSim and OpenAI Gym were implemented and prepared to be used with the Keras library, utilizing Keras-rl [33], which implemented some DRL methods into Python.

The last tool was Tensorflow [34], the Keras backend for the computational activities during the training and testing of the algorithms. Alternatives to Tensorflow are Theano [35], developed by the Montreal Institute for Learning Algorithms (MILA), and the Computational Network Toolkit, better known as CNTK [36], by Microsoft, which is available for Linux and Windows and is used for tools like Cortana.

All of these described tools helped to design the structures of the DQN, SARSA, and A2C algorithms, and then to train and test them. The training times of these algorithms depend on the number of parameters introduced in the DRL networks. Therefore, except for A2C, with one-third, DQN and SARSA had similar numbers. Those from SARSA were higher, challenging DQN, which had fewer parameters, though they were of the same family. Since there were three algorithms, each produced its parameters and apprenticeship ratios. Below is an outline of their structures and explanations for why they were chosen.

The following paragraphs present each algorithm in a general representation, showing the pseudocode. Knowing the structure of the three systems is essential because they collect the data that are later analyzed and compared. 

DQN appeared in 2015 [12]. However, the fragment below, Algorithm 1, was an appropriate adaptation [5] summarizing some of the most often used RL architecture.
**Algorithm 1.** DQN Pseudocode [5]**Input:** the pixels and the game score **Output:** Q action value function (from which we obtain policy and select action) Initialize replay memory *D* Initialize action-value function Q with random weight θ Initialize target action-value function $\hat{Q}$ with weights $\theta^{-}=\theta$ **for** *episode = 1* to *M* **do** Initialize sequence $s_{1}=\left\{x_{1}\right\}$ and preprocessed sequence $\phi_{1}=\phi \left(s_{1}\right)$ **for** *t = 1* to *T* **do**   Following ϵ-greedy policy, select       $a_{t}=\left\{\begin{array}{c} a\;random\;action\;\;\;\;with\;probability\;\epsilon \\ \arg\underset{a}{max}Q\left(\phi \left(s_{t}\right),a;\theta \right)\;\;\;\;\;\;\;\;\;otherwise \end{array}\right.$   Execute action $a_{i}$ in the emulator and observe the reward $r_{t}$ and image $x_{t+1}$   Set $s_{t+1}=s_{t},\;a_{t},\;x_{t+1}$ and preprocess $\phi_{t+1}=\phi \left(s_{t+1}\right)$   Store transition $\left(\phi_{t},a_{t},r_{t},\phi_{t+1}\right)$ in *D*   // experience replay   Sample random minibatch of transitions $\left(\phi_{j},a_{j},r_{j},\phi_{j+1}\right)$ from *D*
   Set $y_{j}=\left\{\begin{array}{c} r_{j}\;\;\;\;\;\;\;\;\;\;\;\;\;if\;episode\;terminates\;at\;step\;j+1\\ y_{j}+\gamma \underset{a^{\prime }}{max}\hat{Q}\left(\phi_{j+1},a^{\prime };\theta^{-}\right)\;\;\;\;\;\;\;\;\;otherwise \end{array}\right.$ Perform a gradient descent step on ${\left(y_{j}+Q\left(\phi_{j},a_{j};\theta \right)\right)}^{2}$ w.r.t the network parameter θ // periodic update of the target network Every *C* steps reset $\hat{Q}=Q$, i.e., set $\theta^{-}=\theta$    **end** **end**

The same code, Algorithm 2, is presented below for SARSA [37]. This is also a reworking [5].
**Algorithm 2.** SARSA Pseudocode [5]**Output:** Q action value function Initialize Q arbitrarily, e.g., to 0 for all states, set action value for terminal states = 0 **for** each episode **do**   initialize state s   **for**
*each step of the episode*, state s is not terminal **do**    a ← action for s derived by Q, e.g., ϵ-greedy    take action a, observe $r,\;s^{\prime }$
    $Q\left(s,a\right)\leftarrow Q\left(s,a\right)+\alpha \left[r+\gamma Q\left(s\prime ,a^{\prime }\right)-Q\left(s,a\right)\;\right]$    $s\leftarrow s^{\prime },\;a\leftarrow a^{\prime }$   **end** **end**

The last algorithm, Algorithm 3, was for the A2C [20], and the extract below was taken from work conducted by J. X. Wang et al. [38].
**Algorithm 3.** A2C Pseudocode [38]// Assume parameter vectors θ and $\theta_{v}$ Initialize step counter t←1 Initialize episode counter E←1 **repeat** Reset gradients: dθ←0 and $d\theta_{v}\leftarrow 0$   $t_{start}=t$   Get state $s_{t}$ **repeat**   Perform $a_{t}$ according to policy $\pi \left(\left.a_{t}\right|s_{t};\theta \right)$   Received reward $r_{t}$ and new state $s_{t+1}$   t←t+1 **until** terminal $s_{t}$ **or**
$t-t_{start}==t_{max}$      $R=\left\{\begin{array}{c} 0\;\;\;\;\;\;\;\;\;\;\;\;\;\;\;\;\;\;\;\;\;\;\;\;\;\;\;\;\;\;\;\;\;\;\;\;\;\;\;\;\;\;\;\;\;\;\;\;\;\;\;\;\;\;\;\;\;\;\;\;\;\;\;\;\;\;\;\;\;\;\;\;\;\;\;\;\;\;\;\;\;\;\;for\;terminal\;s_{t}\\ V\left(s_{t},\theta_{v}\right)\;for\;non-terminal\;s_{t}\;//\;Bootstrap\;from\;last\;state\; \end{array}\right.$
**for** $i\;\in \;\left\{t-1,\ldots ,t_{start}\right\}$ **do**   $R\leftarrow r_{i}+\gamma R$   Accumulate gradients wrt θ:    $d\theta \leftarrow \;d\theta +\nabla_{\theta }\log\pi \left(\left.a_{i}\right|s_{i};\theta \right)\left(R-V\left(s_{i};\theta_{v}\right)\right)+\beta_{e}\partial H\left(\pi \left(\left.a_{i}\right|s_{i};\theta \right)\right)/\partial \theta$   Accumulate gradients wrt $\theta_{v}$: $d\theta_{v}\leftarrow \;d\theta_{v}+\beta_{v}\left(R-V\left(s_{i};\theta_{v}\right)\right)\left(\partial V\left(s_{i};\theta_{v}\right)∕\partial \theta_{v}\right)$ **end** perform an update of θ using dθ and of $\theta_{v}$ using $d\theta_{v}$   E←E+1 **until** $E>E_{max}$


Li defined an apparent association between the problem to solve and the suitability of the algorithm in his work [5]. The work undertaken shows only a part of the association made to exhibit more reasons why SARSA and A2C might outperform DQN. 

These problems, as shown in Table 2, are the ones each algorithm faces. Firstly, they must control the drone to maintain stable flight under external perturbations (such as wind). Secondly, they must compute the data taken after each state–action pair, indicating that fewer cycles achieve superior efficiency. Finally, the behavior when deciding whether to explore or exploit is another marker of the higher or lower performance of the algorithm. However, the specific weight of these issues is likely to be different; therefore, it could seem that the algorithm with a higher percentage of the particular weight with the most positive influencing issue would obtain the best results. Let us also delve deeper into each algorithm to explain its main features. The main code adapts previous modification work [17]; however, our interest lies in the NN structure utilized for the algorithms.

Although the models differ, the number of parameters was close for DQN and SARSA. However, one of the differences in code came up when looking at the number of parameters, which was about 22 million in DQN and SARSA, but in A2C, was around 9 million, slightly less than half. The reason for this is the laptop limitations, which were blocked due to the need for higher capacity. For the records, the processor was an Intel Core i7 7th Generation with an Nvidia GeForce GTX 1070, still throwing a memory allocation error. Therefore, the comparison at this point must be carefully interpreted.

The A2C code is different from the other two in the NN solution, formed by the actor and the critic networks and related to action selection, which depended on the q-values in the case of DQN and SARSA.

The end of the section utilized the data obtained when running the program. An extract below, Table 3, shows what AirSim provided when executed together with AirGym. It must be noted that the data from the table were previously cleaned and ordered, since the result was expressed in comma separated values (CSV).

The episode is the counter, starting when the drone takes off and finishing when it reached the target or collided. The nb_steps is the total number of actions, so at the end of episode 334, the drone had performed 23082 actions. Nb_episode_steps is the number of actions taken during that episode. The *duration*, in seconds, is the time invested in achieving the episode. Lastly, Final_X and Final_Y are coordinates at the collision point or the exact point where the drone found the target, since, according to AirGym settings, the target was made at less than 3 m.

### 2.5. Data Analysis

**Figure 5:**
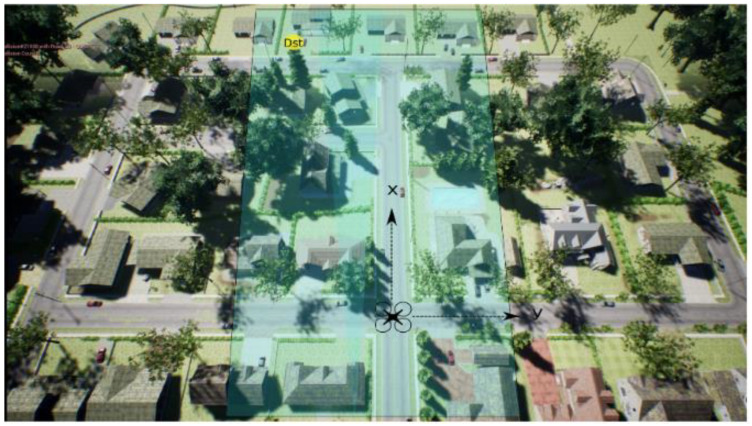
Bird’s eye view of the neighborhood. Drone’s location is A (0,0).

As seen in Figure 5 below, a set of available data provides information on each of the algorithms’ results; thus, it is essential to know the implications of their results in order to make a proper analysis necessary for a later conclusion. Below, the explanation of the meaning of each result begins with the episode and finishes with mean_q.

Episode: A lower final episode counter denotes a higher efficiency. However, this cannot be by chance, meaning that if the drone finds the target randomly or too early in a way that it is not a local minimum, though it was not clearly realized in the training process, it was quickly discarded when tested. When two algorithms were compared in terms of the moment they achieved the target, the one with fewer episodes performed best. The collisions were counted.

Episode_reward: This quantifier provides information about the reward at the end of the episode, which was a cumulative reward. The reward resulted from the time invested, the number of actions performed, and the distance between collisions and the target. Depending on the circumstance causing the episode to end, either a crash or achievement of the target, it provides a different negative or positive value and, thus, a different perspective. Furthermore, if the result is a collision, it provides an idea of how the training process is going.

- A negative value means the collision likely occurred far from the target.
  o A high absolute value says that the drone invested a significant amount of time into randomly traveling from one place to another, flying in the opposite direction to the target, or turning left and right in one place to look around. This might occur during the early exploration phase.
  o A low absolute value means that a short flight occurred, with an early collision with a sudden object during a mature exploration stage.
- A positive value means the collision likely occurred close to the target; in this case, the absolute value would be expected to be low, meaning that the algorithm is improving in the learning process. This case could also be stated at the beginning of the exploration. Finally, if the result is the achievement of the target, the episode reward is a high positive absolute value, meaning that the drone finished training and, thus, avoided obstacles thanks to the learning process and achieved was close to achieving the target.

Nb_steps: This is the total number of actions, so when comparing two algorithms, the one with fewer nb_steps would be the one with the faster learning process. This is also an indicator of the overall performance.

Mean_absolute_error: The difference between the expected value and the obtained value. Like others in the table, this parameter is not expected in all algorithms, so it only helps to understand the process. A later explanation of the results provides more details regarding this.

Loss: This provides information on how comfortably the algorithm modeled the data. High values indicate that it did not model as expected, and low values mean the algorithm modeled it well.

Mean_eps: This is the average epsilon (ε) value for the entire episode after all the actions taken during the episode, either exploration or exploitation. Initially, ε with a value near 1 is an exploration signal. At the end of the learning process, an ε close to zero means that explored has already occurred, thus exploiting the knowledge. Looking at the results, it is visible in the DQN algorithm that it behaved as expected, although its performance will not be discussed here.

Nb_episode_steps: This counter helps in understanding the process of learning. Initially, the algorithm should output random steps per episode while employing an active exploration. The number of steps per episode becomes stable in a more mature phase, thanks to a lower ε, meaning that the algorithm exploits what was learned.

Duration (in seconds of the episode): This must be understood under two cases. The first provides information on a random process, part of the exploration stage in which the collision may occur sooner or later. The second belongs to a mature phase where the algorithm exploits its apprenticeship, and the time decreases as the number of episodes increases. The latter is due to better knowledge of the environment.

Final_X/Final_Y indicate the coordinates, X and Y, where the collision occurred. These values, referenced to time, denote the general behavior of the algorithm, since they shows whether there was a higher or lower exploration vs. exploitation component.

Mean_Q is not a standard indicator for all algorithms, so again, its information is not comparable and only helps in understanding the behavior of the expected reward. Mean_Q is the average of all the Q-values of the entire episode, since there is one value per state–action pair.

Furthermore, it is essential to understand the steps by which to obtain the results that characterize this project and the methods used to obtain them. Firstly, it is crucial to identify the environment, which, by default, uses meters (m) for measuring coordinates and a drone speed of 1 m/s (meters per second), which can be modified. While the drone can freely move in the *x* and *y* axis, the z axis (altitude) was restricted to avoid skipping collisions by traveling over the obstacles.

The distance between start point A (0,0) and endpoint B (35,50) was selected after the first training session using separate coordinates. We noticed that the training time was excessively long.

The evaluation method used measurable parameters, like the learning time, the distance from the collision to the target, the episode time, and the number of episodes. These data provide direct information about the performance of the algorithm.

- Time: A shorter time to reach the target implies better performance than a longer time.
- Distance from collision to B: As collision coordinates progress by traveling closer to B, the algorithm’s learning improves; therefore, comparing, in terms of time, the crash coordinates of a batch in two algorithms, the one traveling a closer distance in a shorter amount of time denotes the better training speed.
- Episode time: A sign of learning is that the episode time should vary as the episode counter increases. However, this does not work for all algorithms in the same way, since, for example, the amount of time could firstly increase and then decrease it, which would mean that the algorithm was exploring at the beginning and exploiting the learning of the exploration afterward. Thus, this counter depends directly on the algorithm.
- The number of episodes: Fewer episodes denote better data/sample efficiency.

## 3. Results

The results section describes the outcomes, considering DQN as the benchmark. Its results are the first to be presented, although the others are referenced. Moreover, since the data are extensive and it is impossible to depict them in a table, graphs are the simplest and the best visual method. Charts and curves were created using R Studio. Table 4 below shows a quick view of the experiments which were performed.

The two points’ (A and B) positions were selected not to be too far apart, which would make the simulation too long, and not too close together, to avoid occasional findings while exploring. The image below, Figure 5, shows a bird’s eye view of the environment, signaling +X and +Y.

**Figure 6:**
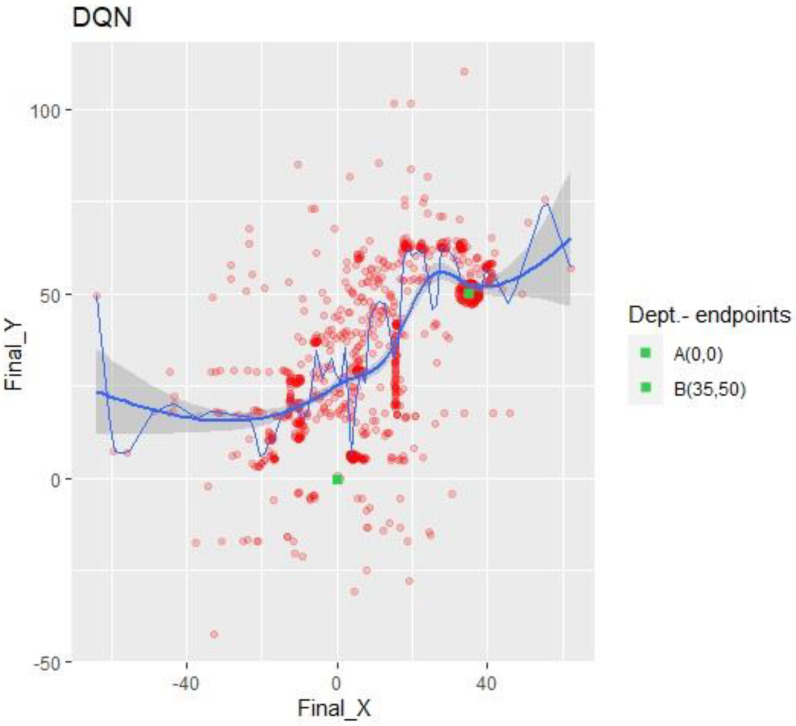
DQN X-Y collision points. Impacts referenced to abscissa and ordinate axes.

The image above has been extracted from the research of G. Muñoz [39] on drone usage for deliveries. Furthermore, a crucial indicator of the accuracy is the drone’s flight speed, set by default to 1 m/s. Next, the individualized graphs for each algorithm show the neighborhood’s collision coordinates; one example is Figure 6. The bold lines show the linear trend and the confidence interval. The confidence interval narrows as the number of collisions increases on the X-axis, denoting an increase in collisions due to a significant concentration of points. The conclusions will be given later. The bold, colored line corresponds to the quantile of 0.5 under additive regression, meaning that half of the collisions occurred above and the other half below. It represents the instant deviation to the linear trends of the data.

The scatter plot shown above, in Figure 6, shows how the drone traveled around the environment under the DQN algorithm. Sometimes, it flew toward the -Y coordinates, but most of its displacements were towards +Y. The X coordinates of the collisions was more equilibrated than those of the Y-axis; however, most collisions occurred in the +X sector. Dividing the graph into four quadrants, the one with the greater number of crashes was (+X, +Y), coinciding with the target quadrant.

The plot’s narrowest zone coincides with X = 35 ± 3; target B was considered to be reached when the drone arrived within a circle of 3 m around the coordinates (35, 50), as established in the AirGym code for the world’s settings.

**Figure 7:**
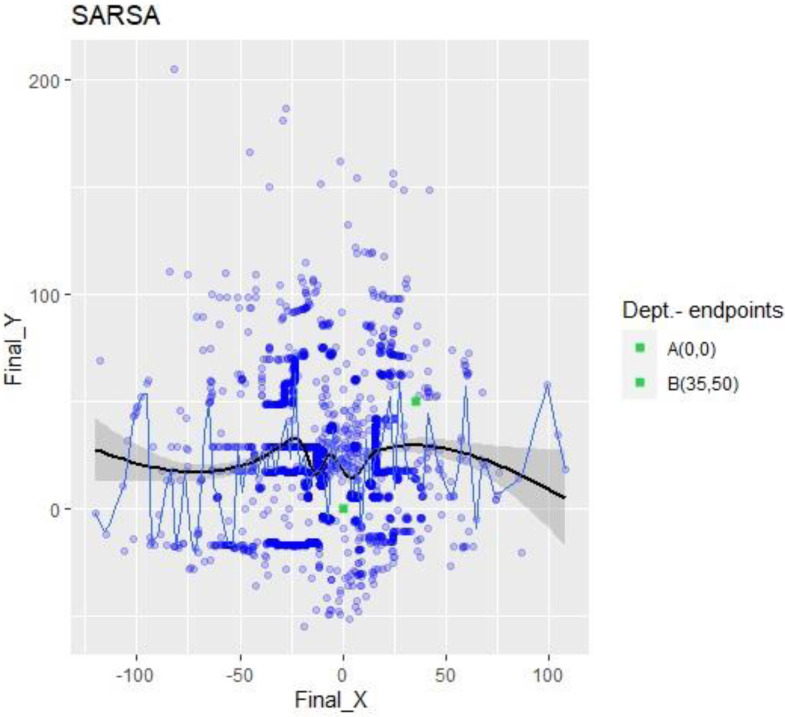
SARSA X-Y collision points. Impacts referenced to abscissa and ordinate axes.

The scatter plot of SARSA collisions, Figure 7, shows drone crashes all over the environment. There is no uniform pattern, and all points are distributed around the map, which is visible because the linear trend and the median (quantile = 0.5) show no specific tendency or convergency to any point.

**Figure 8:**
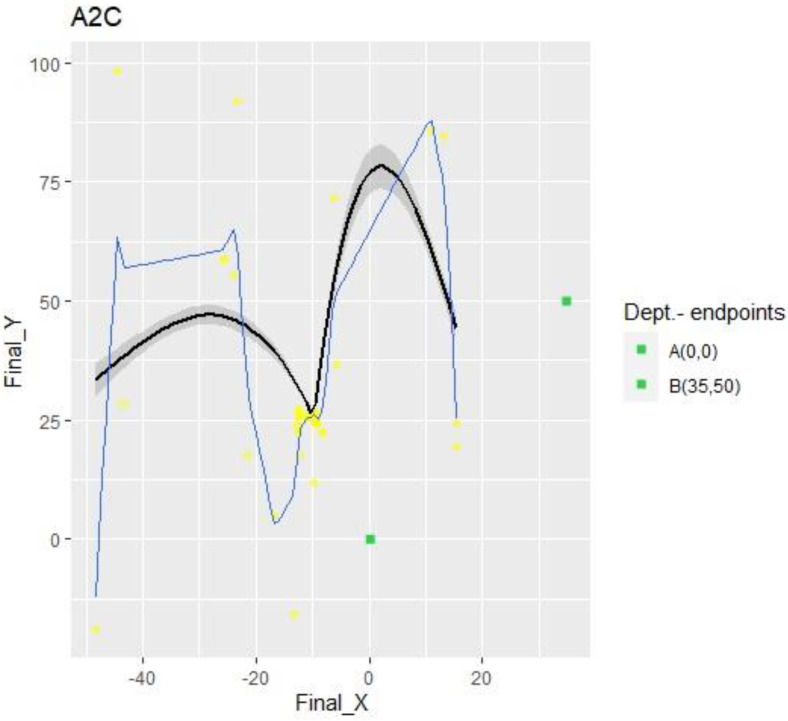
A2C X-Y collision points. Impacts referenced to abscissa and ordinate axes.

However, the most unexpected result came from the A2C algorithm. The graph below, Figure 8, shows that the drone fell into a local maximum after one-fifth of the episodes, meaning more than 35 K out of 150 K actions.

**Figure 9:**
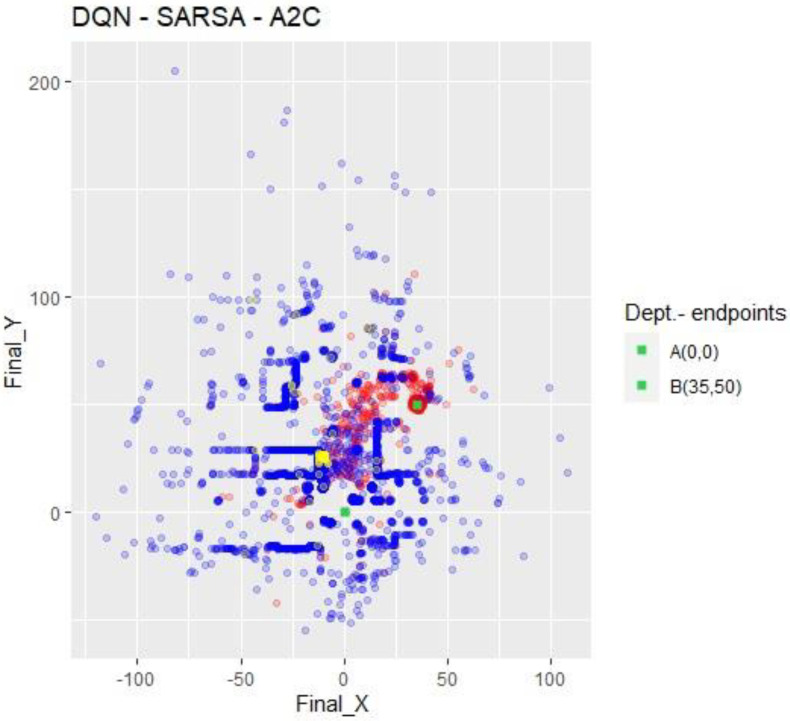
All X-Y collision points. Impacts referenced to abscissa and ordinate axes. Colors are conserved from previous corresponding Figures.

Figure 9 combines the previous three. In red are DQN’s collisions; in blue, SARSA’s; and in yellow, A2C’s. Due to the high dispersion, SARSA’s collisions abstractly represent the top view of the “neighborhood”. The red dots show a particular evolution of the collisions of DQN towards target B. Finally, the yellow dots show how short the flight of the drone was when performed under the A2C NN.

**Figure 10:**
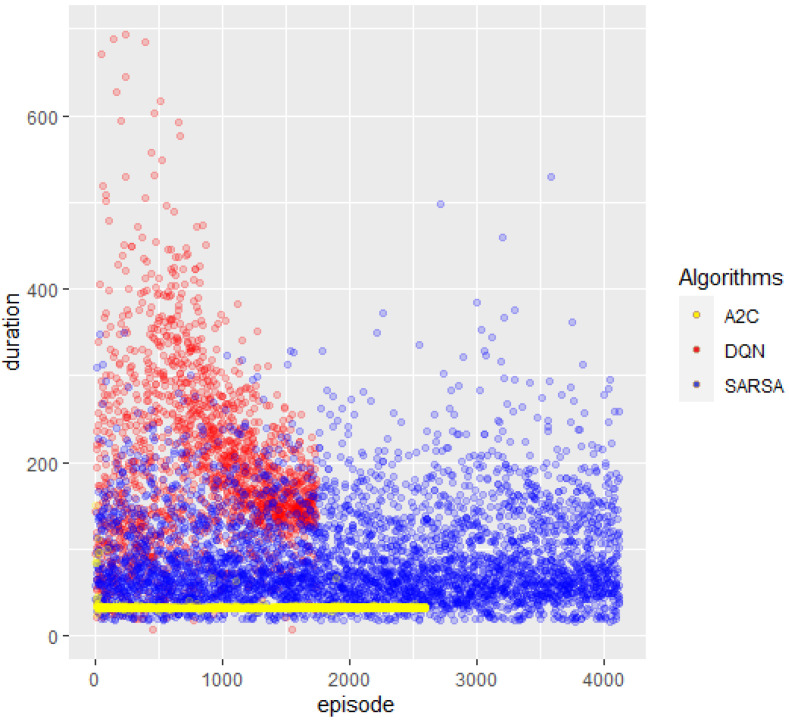
Episode duration. Abscissa references the episode counter; ordinates, the reference time.

The previously explained indicators were referenced to the time, considering time as the episode number, since as time passes, actions occur and episodes increase; this is the direct relationship between time and the episode number. Figure 10, below, shows the duration of the episode, with the colors assigned as earlier for the previous graph.

Looking closely at the DQN (red) episodes, they seem to have had a concise duration at the beginning mixed with some long-duration ones; however, as the episode number progressed, the episode’s duration suddenly rose smoothly to a tendency of length reduction as time passed. In blue, the SARSA network shows a regular predisposition of a superior percentage of short-duration episodes mixed with random medium and long-duration ones, a trend which was maintained until the end. Finally, in yellow, it is shown that the A2C algorithm initially deployed some long-duration episodes, but especially early on in the number of episodes, it remained stuck in a continuous, same-extent episode.

Except for A2C, which fell into a local maximum (stuck in a continuous same-extent episode) and cut the training after performing 2600 episodes and 35 K actions, the other two completed the 150 K actions established as a baseline for the process—more than 4 K episodes for SARSA and less than 2 K for DQN.

**Figure 11:**
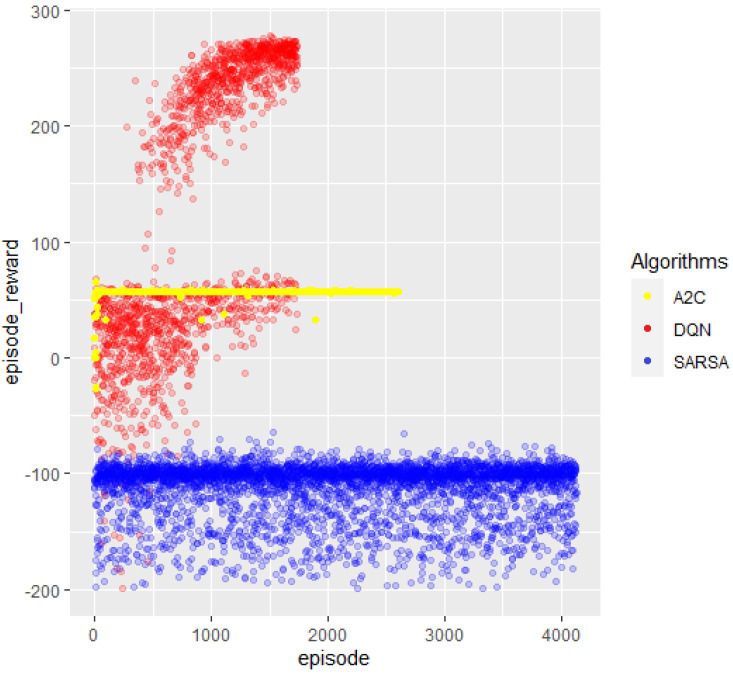
Episode reward. Abscissa references the episode counter, and ordinates reference the reward.

The last functional chart with which to compare the three algorithms is in Figure 11, which displays the episode reward vs. the episode counter. The colors are the same as those used for the previous descriptions. We analyzed DQN’s highest rewards knowing it is the only algorithm that reached the target. The first thing to notice is that, in many cases, it reached a higher action reward than 275, since every time there was a collision, it received a deduction of −100. Hence, those values close to 200 seem to have reached almost 300 before the crash. Secondly, looking at the distribution of time, the beginning spots were mainly concentrated on an episode reward value region between zero and sixty, with a lower congregation around lower reward rates. As time passes, the concentration zone moved to higher reward values and abandoned the negative ones. For the highest episodes, most spots were concentrated on reward estimates of 280.

SARSA’s distribution of reward spots over time was regular, without changes. There was a high intensity of around −100, which decreased for lower reward ratios. Finally, A2C dissemination did not exist; it was linear for rewards equal to 57.

Table 5, below, summarizes the results presented in the text above, indicating with a ✓ where it performed as expected.

## 4. Discussion

This section expands on the relationship between the charts deployed in Section 3 and the expectations for each algorithm, establishing a comparison between them. It also compares the results obtained for DQN and questions it by comparing it with previous works.

Several graphs are presented in Section 3, which are apparently interpretable separately; however, correlations and implications oblige us to understand them when they are combined. For readiness and simplicity, it is important to remember that the comparison between the three networks is intended to answer the question of which had the best performance in terms of reaching the target, considering the exact departure coordinates for a maximum number of total actions equal to 150 K. In the case of A2C, the full parameters were those from the actor plus those from the critic.

DQN excels at the exploration–exploitation trade-off, implying that it finds the best combination between the two alternatives to reduce “effort”. With around 22 million parameters and a maximum of 150 K actions, it was the only algorithm that reached the target. This suggests that SARSA and A2C need more time (actions) or more parameters to accomplish the same goal, indicating their low performance for this task. However, when considering the data sample efficiency (measurement of the algorithm’s performance with low data acquisition), A2C should have excelled over the other two. This was perceived from Figure 9 and Figure 10, since it found a local maximum at a very early stage.

Nevertheless, discovering an early local maximum and still not reaching the global maximum (target B) constitutes an unsatisfactory overall performance. However, it shows an attitude that the other two algorithms did not exhibit, which gave us the idea that A2C, under other comparison conditions, could overcome DQN efficiency, since DQN took a long time to reach a maximum and A2C found a maximum (local) at an early stage (data sample efficiency). A swift response regarding how to solve this problem is to give the actor a percentage of the DQN feature—the exploration–exploitation trade-off.

Finally, the last factor, the control, is a characteristic of SARSA and A2C. Control stands for attitude, altitude, and position. The altitude is fixed; nevertheless, attitude and position are variables. The position results from the attitude and the altitude, so the attitude must provide an explanation referring to hovering, takeoff, landing, and collision avoidance maneuvers. AirSim implements hovering, takeoff, and landing by itself; thus, collision avoidance is the only maneuver to face and, consequently, explain. Therefore, to link this with the previous paragraph, on the one hand, A2C exhibited extraordinary control because it found a way to avoid obstacles quickly and reach the said “local maximum,” but needed to undergo a few crashes to find that “good” way/path.

On the other hand, the SARSA perspective is different because it explored the entire environment without implementing exploitation, which means that it crashed continuously. Interestingly, it never hit the same point twice, implying its positive attitude toward collision avoidance. Control, referred to as collision avoidance, was not seen in DQN, since most of its collisions were in the same zone due to its exploration–exploitation trade-off. Although only DQN reached point B, the importance of the prior results is due to the fact that the algorithms did what they were supposed to do independently of the efficiency. Thus, different features weigh differently in performance, influencing the behavior differently.

**Figure 12:**
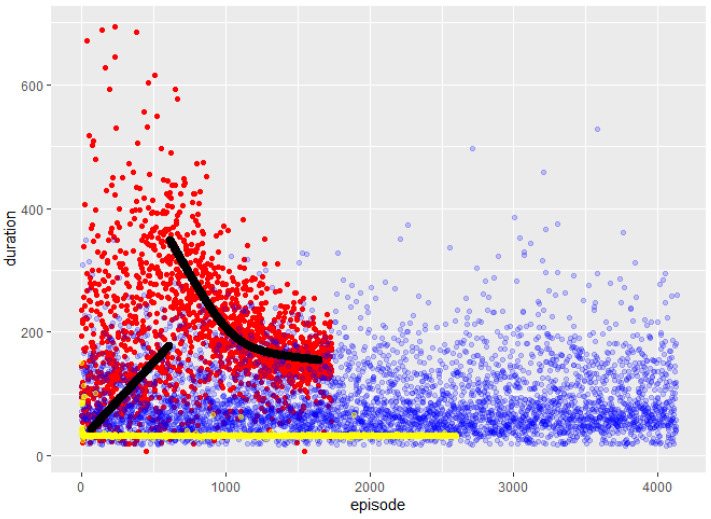
Episode duration. Abscissa references the episode counter; ordinates, the reference time. The black lines show the apprenticeship tendency. Colors correspond to DQN, SARSA and A2C as used previously. Black lines show the apprenticeship tendency of DQN.

From Figure 12, it follows that DQN started its learning with a concise duration, meaning that most of the crashes occurred close to the departure zone, but as time passed, the length of the episodes increased with a positive slope, which can be interpreted as a longer traveled distance. This is also part of the exploration process. Suddenly, the behavior of DQN changed, and the extent was augmented substantially, indicating that the exploitation process had begun. This process was not linear, since the learning procedure depends on the NN’s structure. The inference is that the greedy policy increased the reward by decreasing the time wasted in each episode. The beginning of the exploration (indicated by the curve line) had a superior duration because it had only an “idea” of the proper direction of the target. Over time, the direction was polished, implying a shorter episode duration due to the need to execute fewer actions. The entirety of the DQN spots representation was as expected for the project when considering the exploration–exploitation trade-off characteristic.

The durations of the SARSA episodes revealed a continuous exploration, which is understandable, since this algorithm did not have a greedy policy to combine with exploitation. The environment contained plenty of different objects, and there were no similitudes between zones to allow for a short apprenticeship. Furthermore, an algorithm that excels at control is meant to travel until it knows the whole atmosphere, learning not to collide in the same place twice. This observation assumes that the time (150 K action) specified for the training was too short for SARSA, and that it would have needed more episodes to “study” the neighborhood. This interpretation is reinforced in Figure 11, where it can be observed that SARSA changed flying directions radically after a collision, meaning it was “thinking and learning” to avoid falling twice in the same error (collision coordinates). According to its characteristics, it can be considered that its performance was not that which was expected overall for the selected application.

The result revealed by the A2C was very different; it fell into a local maximum and never found a way to escape from it. However, the most exciting aspect of this result is how quickly it found the local maximum. This is because, observing Figure 11 again, it is noticeable that the algorithm discovered how to maximize the function within very few episodes. Furthermore, it is essential to note that A2C accomplished this with less than half of the parameters that DQN and SARSA used. Being so efficient in so little time indicates that A2C has a potent network that is capable of overcoming the current results with the proper hardware when working under established conditions.

Although A2C could not escape the local maximum, some spots show that it attempted to consider other alternatives several times, resulting from the lower quantity of parameters compared to DQN or SARSA. These tries show longer durations and more actions with lower total rewards than those in the local maximum; thus, A2C was not able to leave the local maximum behind.

The second paragraph of this section indicated that each algorithm met its expectations. Although DQN is the benchmark and the only one that reached the target, the three algorithms exhibited excellent behavior in control, data sample efficiency, and exploration vs. exploitation trade-off, as indicated in Table 2. However, one purpose of the project was to verify how well DQN could perform in this environment, and whether the other two, SARSA and A2C, could prevail over it, that is, to question the benchmark and its suitability. With the selection of the number of layers and parameters, it is clear that DQN remains undisputed, which means that for this particular application, the features from SARSA and A2C did not match well. This does not suggest that they are the worst algorithms, but indicates that the exploration–exploitation trade-off feature has a more decisive influence on this environment.

**Figure 13:**
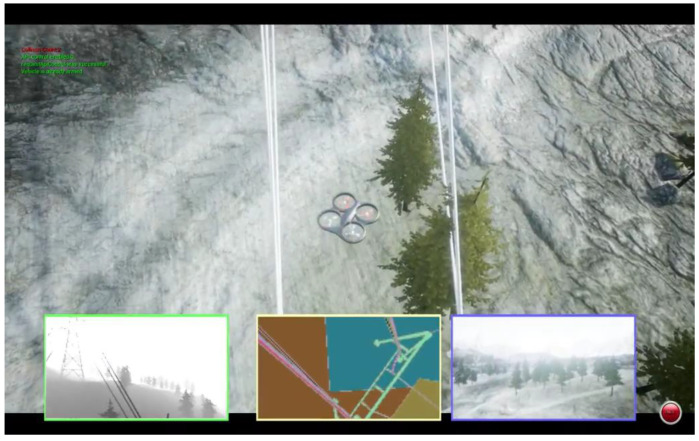
RL DQN powerline surveillance [18]. Distances between the object matter under study and the rest of the environment were excellent; therefore, it was easier for the algorithm to isolate the powerlines from the other objects.

**Figure 14:**
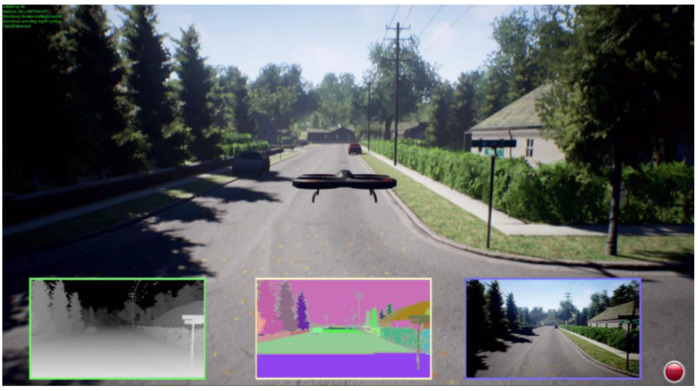
Neighborhood created by Microsoft in [18]. The experiment used the miniature image in black and white in the left-low corner, employing aa stereo camera.

**Figure 15:**
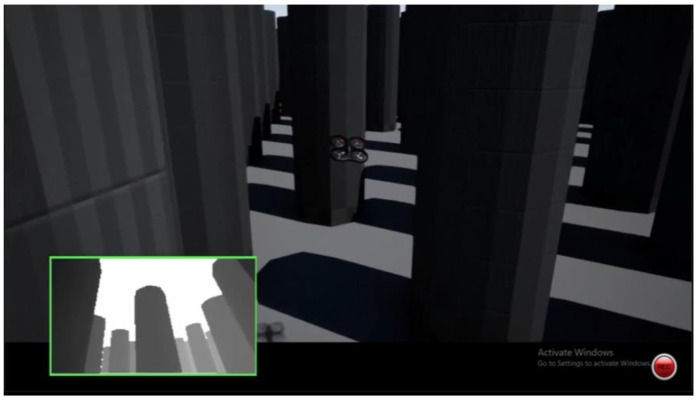
The environment used by Kjell [17]. Columns and distances between them were the same for the whole environment, making them more accessible for learning by pattern recognition using RL.

The Microsoft AirSim team used DQN [18] for a powerline follow, Figure 13, to help simplify human labor; the difference in their research approach was the simplicity of the object in its looks, which, due to heights, was differentiable and, therefore, easier to learn for the NN. This means that, even in a very complex overall environment, the discrimination between objects is faster. Furthermore, the same Microsoft Team, in [18], used DQN in the neighborhood world to teach a car to travel on the roads, Figure 14. This was more straightforward, since the number of different objects was not as high as when flying. Kjell used DQN with a satisfactory test result [32], Figure 15. However, his environment, formed by many columns of the same diameter and height in the background with standard color, was more straightforward than the “neighborhood” used in this dissertation. The reason for this is that the NN only needed to learn to identify one object, which looked the same from any angle. In the neighborhood which was used, everything, including cars, trees, leaves, traffic signals, and moving deer, looked different from distinct perspectives, and also, due to shadows, the light contrasts multiplied the complexity exponentially.

In conjunction with the discovery made in this work that these algorithms did not perform correctly in the test phase, the two examples above might lead us to think that DQN would have limitations in cases where the environment has plenty of distinct objects to be analyzed. On the contrary, the efficiency of the data sample from A2C could meet the expectations for such complex conditions. There were certain constraints in this work, and the most important was the willingness to make a comparison between exactly equal requirements. 

The observation of three images’ shows that, using stereo camera sight (subpicture in the left-down corner squared in green), the differences were appreciable when comparing the first and third with the second. In the third picture, the obstacles all look all the same; in the first, the closest objects are the power cables, which also look identical. However, once the drone flies through the inner gardens in the second picture, everything is unique, even from a distinct point.

Therefore, the limitation of this work is explicit, because the results depend enormously on the chosen environment and the number of layers and parameters used. As has already been said, under the conditions of a particular chosen environment with a specific number of neurons, DQN reached the target. In contrast, the others did not, but this does not suggest that the results would be equal under other, more straightforward or demanding conditions.

## 5. Conclusions

The section provides an overview of the three algorithms’ performances and their suitability for this particular purpose.

This research allowed us to acquire knowledge regarding the proper choice of a suitable family of algorithms for assessing a UAV’s travel patterns in a previously unknown area, where it is required to find the path from the starting point to the endpoint without crashing, avoiding the obstacles encountered. Below, the main ideas extracted from the comparative work analysis are listed.

This research contributes to the knowledge that SARSA is not an appropriate RL algorithm for systems where the observation space differs for the same object when observed from a different perspective. This is because it tends to learn the entire environment before undertaking a job, and, therefore, needs an exhaustive amount of time for it. Thus, we conclude that the control feature should not be considered for this task. Regarding A2C, the conclusion was more satisfactory because proper hardware and good network modeling saved time and computing resources on in the accomplishment of the job. DQN did not perform as efficiently as expected in a complicated system, since the endpoint was reached only during the learning process. Therefore, this algorithm should only be used for more accessible or medium-difficulty environments, like the columns environment.

These results will open new doors for several lines of investigation, some concerning the DQN and others, the A2C. Regarding DQN, it is interesting to find its limits when considering more complex environments. It is crucial to seek answers in order to understand whether the poor performance during the testing was due to a lack of parameters for the environment or the NN itself. It is not evident that increasing the number of neurons would help to solve the stated issue; however, investigating and comparing distinct behaviors is a great idea when the number of parameters for the neighborhood system is progressively raised. Different results might arise if checking is conducted for a fixed number of neurons and a parceled environment, and if training is intended for increasing plant surfaces until it is clear which extension is too large for DQN.

Regarding A2C, the first step is to find the structure that will cause it not to fall into a local maximum, which was not the purpose of this development. Once the net’s shape is found, performing some evaluations like those described below will be a useful prospect. One proposed exercise is to determine the minimum number of neurons needed to achieve the task in this work and to establish the performance per neuron of the network when faced with DQN, which might allow us to consider that A2C could become a benchmark in complex systems. A second strategy is to compare the time needed to achieve the task with the same number of parameters as DQN in order to guess the learning speed in challenging environments, providing another reference with which to establish a new benchmark.

## Figures and Tables

**Table 1:** Feature–algorithm cross table. Transfer-learning-oriented approach (TLOA); learning shared representations for value functions (LSRVF), progressive neural networks (PNN), asynchronous advantage actor–critic (A3C), advantage actor–critic (A2C).

Feature	TLOA	LSRVF	PNN	PathNet	Policy Distillation	Actor-Mimic	A3C	A2C
Scalability	x	x	x	x	x	x	x	x
Distraction Dilemma	x	x		x		x		
Partial Observability			x	x			x	x
Effective Exploration		x	x			x	x	x
Catastrophic Forgetting			x	x			x	x
Negative Transfer Learning				x			x	x

The x means that the algorithm owns the property.

**Table 2:** Issue–algorithm association.

ISSUE	DQN	SARSA	A2C
Control		x	x
Data/Sample efficiency			x
Exploration-exploitation trade-off	x		

**Table 3:** Fragment of data obtained from the simulation.

EPISODE	EPISODE_REWARD	NB_STEPS	MEAN_ABSOLUTE_ERROR	LOSS	MEAN_EPS	NB_EPISODE_STEPS	DURATION	FINAL_X	FINAL_Y	MEAN_Q
334	51.79016999	23,082	431.389453	3839.994081	0.792433	37	102.79076	15.28926086	25.0212822	650.9491297
335	17.6551504	23,135	429.617013	4007.002654	0.792028	53	139.3530722	−10.80948925	25.70606232	647.0104923
336	26.32453733	23,175	429.4691521	4223.102121	0.7916095	40	104.474514	−12.27823734	21.01215553	646.9784271
337	46.15792278	23,194	433.0540081	5953.84613	0.791344	19	54.24276996	−9.695857048	15.28050995	650.7867881
338	−48.79513635	23,342	429.3128794	4320.594986	0.7905925	148	375.640465	24.12601662	71.77313232	648.1717076
339	42.0800166	23,365	433.8253744	3208.099129	0.789823	23	64.18440485	−9.896894455	14.42659092	661.6635954
340	10.33699335	23,454	429.1657711	4372.652276	0.789319	89	226.855993	18.81990814	63.92234421	647.2763624

**Table 4:** Summary table of the experiments and their analyses.

Experiment	DQN	SARSA	A2C
X-Y collision point	Figure 6	Figure 7	Figure 8
Duration—episode	Figure 10	Figure 10	Figure 10
Episode reward—episode	Figure 11	Figure 11	Figure 11

**Table 5:** Summary table of the results.

Experiment	DQN	SARSA	A2C
X-Y collision point	✓	✓	
Duration—episode	✓		✓
Episode reward—episode	✓		

## Data Availability

Data can be made available to interested readers by contacting the authors.
